# Dancing Leadless Pacemaker: Dislodgement of a Ventricular Screw‐In Leadless Pacemaker Driven by Severe Tricuspid Regurgitation

**DOI:** 10.1002/joa3.70335

**Published:** 2026-04-07

**Authors:** Kazuki Shimojo, Itsuro Morishima, Yasunori Kanzaki

**Affiliations:** ^1^ Department of Cardiology Ogaki Municipal Hospital Ogaki Japan

**Keywords:** device dislodgement, device retrieval, leadless pacemaker, tricuspid regurgitation

## Abstract

Following successful right ventricular septal fixation, the leadless pacemaker abruptly dislodged under the influence of a severe tricuspid regurgitant jet. Alternating regurgitant and inferior vena cava inflow forces produced a characteristic to‐and‐fro oscillation between the right atrium and inferior vena cava, with eventual successful retrieval after migration into the distal coronary sinus.

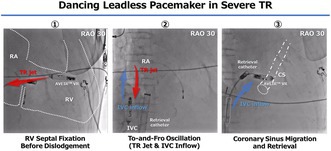

## 1 Introduction

Leadless pacemakers have improved safety by eliminating lead‐ and pocket‐related complications [1]. However, although infrequent, device dislodgement remains a serious event [2, 3]. Post‐implantation tricuspid regurgitation (TR) has been recognized as an important clinical issue [4]. In this case, pre‐existing severe TR with marked right atrial (RA) enlargement likely contributed to device instability through a combination of hemodynamic stress and structural remodeling.

## 2 Case Report

An 81‐year‐old man with severe mitral stenosis (valve area 0.86 cm^2^), severe TR (TR pressure gradient 41.0 mmHg), biatrial and inferior vena cava (IVC) enlargement (IVC diameter 25.4 mm), chronic atrial fibrillation, recurrent cerebral embolism, thrombus in the left atrial appendage, and preserved left ventricular ejection fraction was referred for pacemaker implantation because of complete atrioventricular block. Given his frailty (Clinical Frailty Scale 6), a helix‐fixation leadless pacemaker, AVEIR VR (Abbott, Chicago, IL) was selected.

**FIGURE 1:**
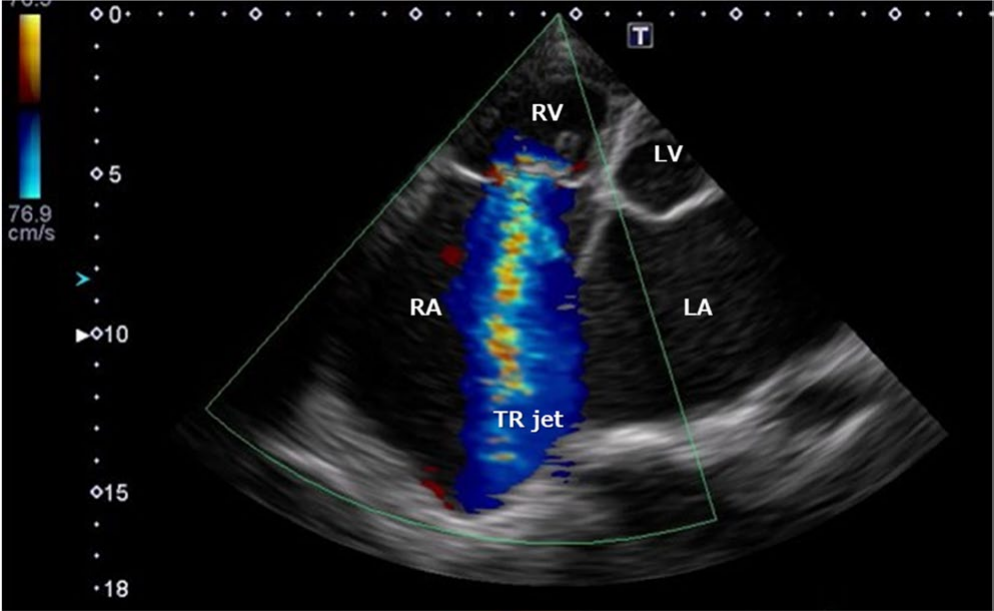
Transthoracic echocardiography before implantation showing a severe TR jet directed toward the septal side of RA. Transthoracic echocardiography demonstrated severe TR with a high‐velocity jet directed toward the septal side of markedly dilated RA, suggesting significant hemodynamic stress on the septal wall prior to implantation. LA, left atrium; LV, left ventricle; RA, right atrium; RV, right ventricle; TR, tricuspid regurgitation.

**FIGURE 2:**
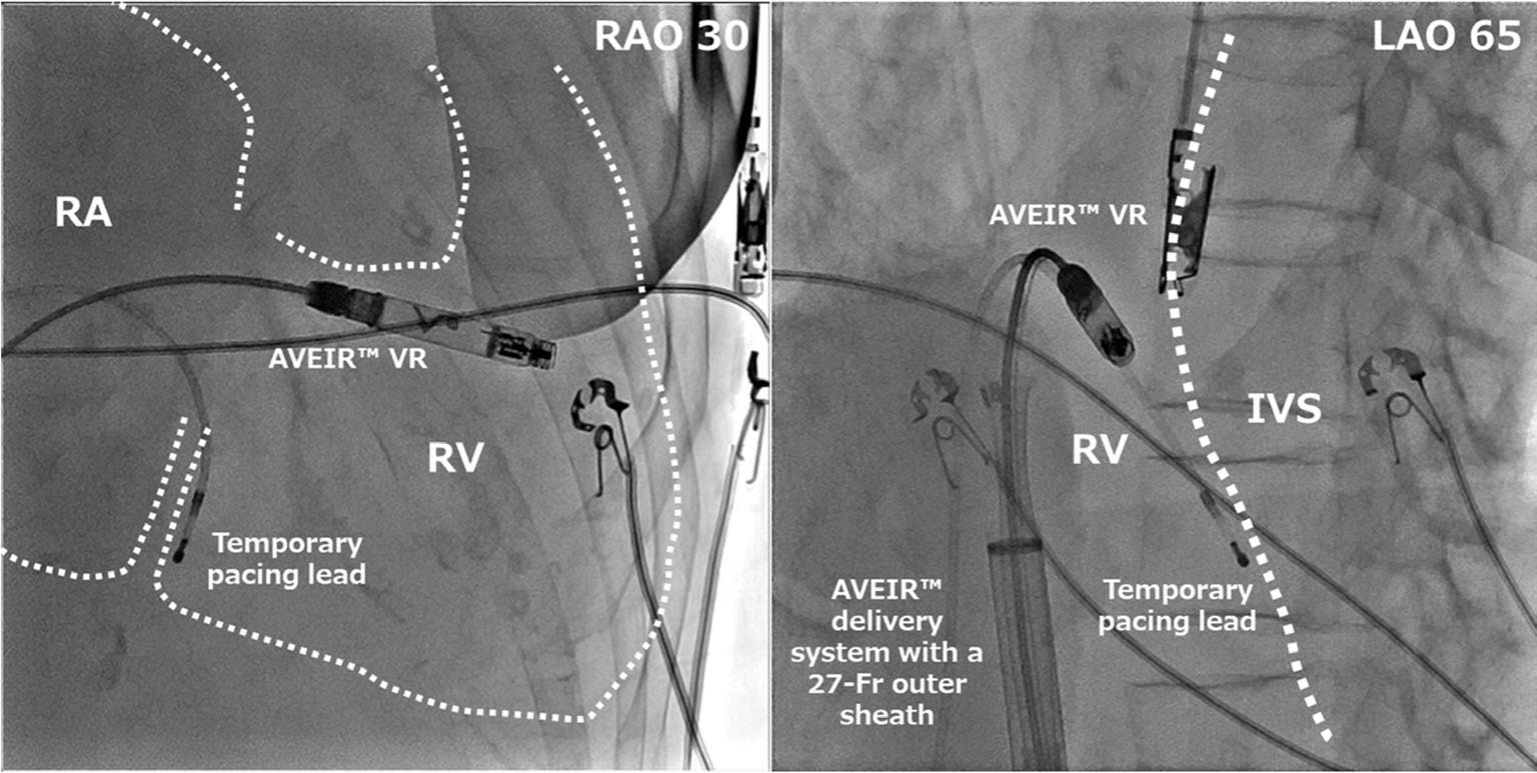
Fluoroscopic image during the initial implantation showing AVEIR VR positioned on the RV septum. Fluoroscopy confirmed appropriate positioning of the AVEIR VR leadless pacemaker on the mid‐RV septum with stable fixation and acceptable electrical parameters immediately after deployment. IVS, intraventricular septum; RA, right atrium; RV, right ventricle.

Due to massive TR and marked RA enlargement (Figure 1), deployment onto the right ventricular (RV) septum was difficult. Repeated deployment attempts were unsuccessful due to lack of impedance rise, high pacing thresholds, instability during deflection testing, and mechanically induced ventricular tachycardia. After multiple repositioning attempts, stable lead fixation was eventually achieved in the high septal region with acceptable parameters: impedance increased from 380 Ω pre‐deployment to 500 Ω post‐deployment, sensing was 3.2 mV, and pacing threshold was 1.25 V at 0.24 ms. Adequate mechanical fixation was confirmed by a successful deflection test (Movie S1A and Figure 2). An increase in injury current was also observed at the time of screw fixation, suggesting myocardial engagement. No excessive swing motion (approximately > 45°) was observed at the final implantation site prior to release.

**FIGURE 3:**
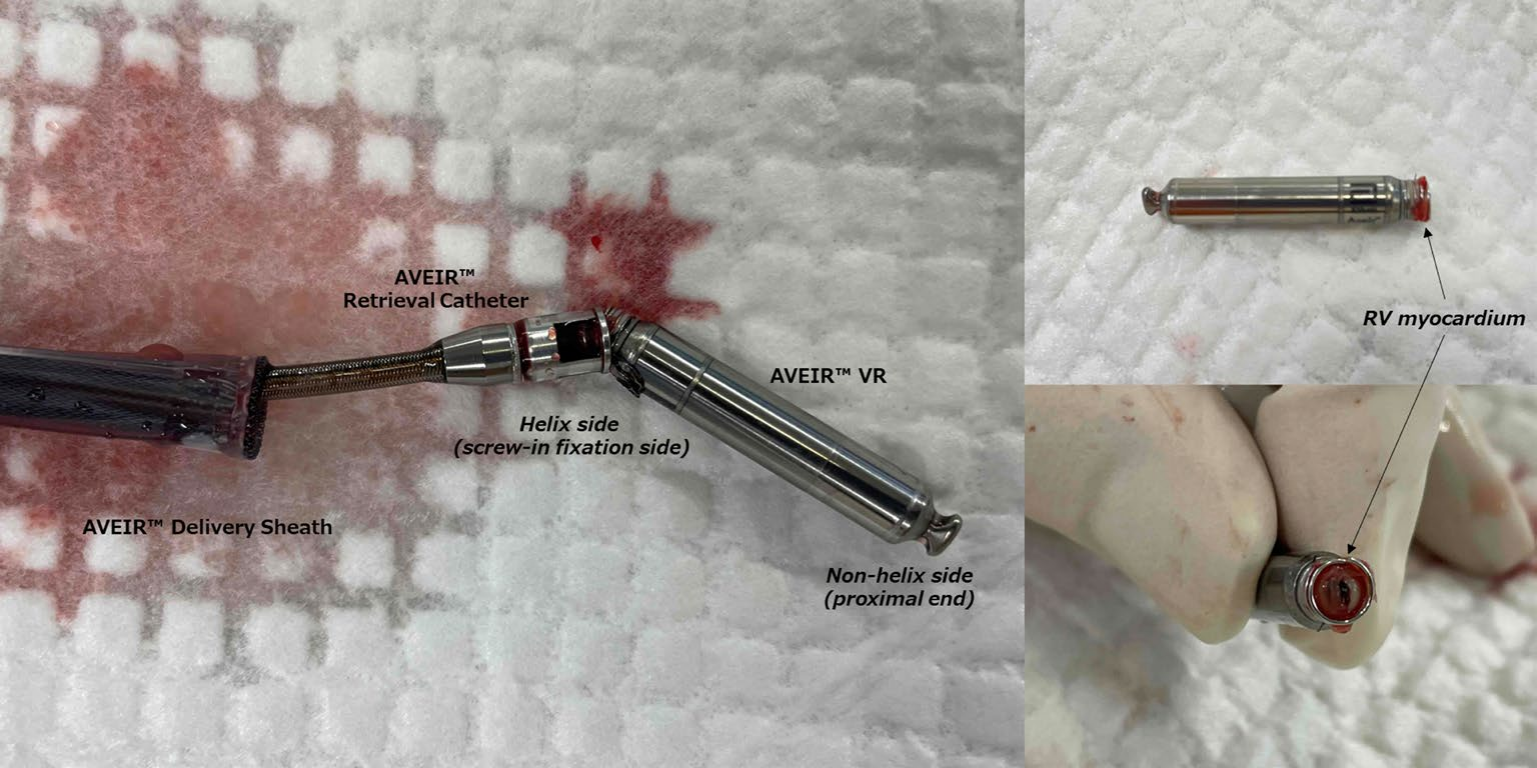
Images of the AVEIR VR leadless pacemaker after retrieval. The device was grasped at the helix, with the residual RV myocardial tissue attached to the helix. RV, right ventricular.

However, shortly after detachment from the delivery cable, the AVEIR VR abruptly dislodged from the RV septum and was driven retrogradely by the TR jet into the RA (Movie S1B). The device then exhibited a striking oscillatory motion, shuttling between the RA and IVC in synchrony with the pulsatile tricuspid regurgitant flow (Movie S2). Retrieval attempts within the RA were initially performed using the dedicated AVEIR Retrieval Catheter (Abbott Laboratories, Chicago, IL, USA), introduced through a 27‐Fr sheath and incorporating a triloop snare mechanism; however, these attempts were unsuccessful because of the device's high‐velocity oscillatory movement. An additional GooseNeck‐type snare was therefore introduced to temporarily stabilize the oscillating device by grasping it with a snare. During this maneuver, and likely influenced by the severe TR flow, the device migrated into the coronary sinus. In that position, the screw end became more accessible. The pacemaker was subsequently captured and retrieved using the triloop snare (Movie S3). Owing to the reverse orientation of the device within the distal coronary sinus, where the confined anatomical space limited device manipulation and reorientation, the snare engaged the fixation helix rather than the proximal retrieval feature (Figure 3). We acknowledge that retrieval in this configuration carries a potential risk of damage to the helix, snare system, or protective sleeve. However, given the technical difficulty of the initial implantation and the decision not to pursue reimplantation of a leadless pacemaker, priority was placed on safe and timely retrieval.

A screw‐in VVI pacemaker was subsequently implanted via the left subclavian vein. Post‐implant parameters were optimal, with impedance of 460 Ω, pacing threshold of 0.5 V at 0.4 ms, and R‐wave amplitude of 2.5 mV, indicating stable fixation and adequate sensing. The postoperative course was uneventful, and no embolic or valvular complications occurred.

## 3 Discussion

This case illustrates a multifactorial mechanism of leadless pacemaker dislodgement involving both severe TR–related hemodynamic forces and RA structural remodeling. In this patient, the severe TR jet was directed toward the septal region, where intermittent high‐velocity regurgitant flow may have altered local septal mechanics adjacent to the basal ventricular septum at the site of AVEIR VR anchoring. Concomitantly, marked RA enlargement and septal distortion may impair effective trabecular engagement, thereby compromising anchoring stability. Together, these hemodynamic and anatomical factors likely acted synergistically to predispose the device to early dislodgement, with neither factor alone fully explaining the event. The presence of attached myocardial tissue on the retrieved helix further supports that initial screw engagement had been achieved, suggesting that the event was unlikely due to simple unscrewing alone.

Once detached, the same TR jet propelled the pacemaker backward into the IVC during systole, while diastolic inflow drew it back into the RA, producing the characteristic to‐and‐fro oscillatory motion observed fluoroscopically (Movie S2). This pattern represents a direct mechanical consequence of severe TR that has not been previously described in cases of device embolization. Notably, this phenomenon illustrates that severe TR can generate alternating regurgitant and inflow forces of sufficient magnitude to actively mobilize a dislodged leadless pacemaker between the IVC and RA. Eventually, the dislodged device migrated into the distal coronary sinus, where the confined anatomical space limited device manipulation and reorientation. This migration was likely facilitated by atrial flow reversal, similar to a previously described AVEIR VR migration case [5].

Most reported dislodgements are attributed to technical or fixation‐related factors [2]. In contrast, this case highlights the potential role of hemodynamic stress in the setting of severe TR and RA remodeling. Rather than a single dominant mechanism, the event likely resulted from an interaction between structural distortion and acute regurgitant forces. Comprehensive pre‐implant echocardiographic assessment of TR severity, jet orientation, and RA geometry may therefore be warranted. Dynamic imaging remains essential for understanding device behavior and guiding safe retrieval.

## 4 Conclusions

Severe TR, particularly in the setting of advanced RA remodeling, may exert hemodynamic forces that could contribute to leadless pacemaker instability. Recognition of this potential mechanism may help inform deployment strategies and post‐implant monitoring in patients with advanced valvular disease.

## Author Contributions

Dr. Kazuki Shimojo conceived the case report and drafted the manuscript. Dr. Itsuro Morishima performed the implantation procedure and was responsible for clinical oversight of the case, conception of the case report, manuscript revision, and approval of the final manuscript. Dr. Yasunori Kanzaki provided critical revision of the manuscript.

## Funding

The authors have nothing to report.

## Ethics Statement

This case report was conducted in accordance with the ethical principles of the Declaration of Helsinki (2024).

## Consent

Written informed consent was obtained from the patient for publication of this case report and any accompanying images.

## Conflicts of Interest

The authors declare no conflicts of interest.

## Supporting information


**Movie S1:** (A) Deployment of the AVEIR VR leadless pacemaker in the right ventricular septum. A deflection test confirms stable fixation of the device without evidence of instability or dislodgement. (B) Acute device dislodgement after release from tether mode. Shortly after tether release following successful implantation, the AVEIR device is abruptly displaced from the right ventricular septum by a severe tricuspid regurgitant jet.

**Movie S2:** To‐and‐fro oscillatory motion of the dislodged device between the right atrium and inferior vena cava. The device moves bidirectionally, likely driven by alternating tricuspid regurgitant flow and inferior vena caval inflow.

**Movie S3:** Percutaneous retrieval of the dislodged AVEIR device. Using a dedicated retrieval catheter and sheath, the pacemaker lodged in the distal coronary sinus is successfully grasped from the helix side and retrieved.

## Data Availability

Data are available from the corresponding author upon reasonable request.
